# Supplementary material for: Spatial distribution of Culex mosquitoes across England and Wales, July 2023
Source: Parasit Vectors. 2025 Aug 6;18:337. doi: 10.1186/s13071-025-06975-w (PMC12329923; doi:10.1186/s13071-025-06975-w)
Supplement: Supplementary file 1 — Additional file 1. [file 13071_2025_6975_MOESM1_ESM.pdf]

Supplementary information

**Mosquito species or biotype**	**Number of adults caught during July 2023**
Culex *pipiens* complex only	79
Culex *pipiens* biotype *pipiens*	1,478
Culex *pipiens* biotype *molestus*	5
Culex *pipiens/molestus* hybrids	22
Culex *torrentium*	38
Culex *modestus*	0
Aedes cantans/annulipes	16
Aedes cinereus	2
Aedes detritus	6
Anopheles atroparvus	1
Anopheles claviger	8
Anopheles maculipennis s.l.	16
Anopheles plumbeus	19
Coquillettidia richiardii	82
Culiseta annulata	97
Culiseta litorea	1
Culiseta morsitans	3

Table S1. The numbers of *Culex* and non-*Culex* mosquito species identified by molecular and morphological means during the July 2023 field survey.

Presence of four *Culex* species across England and Wales

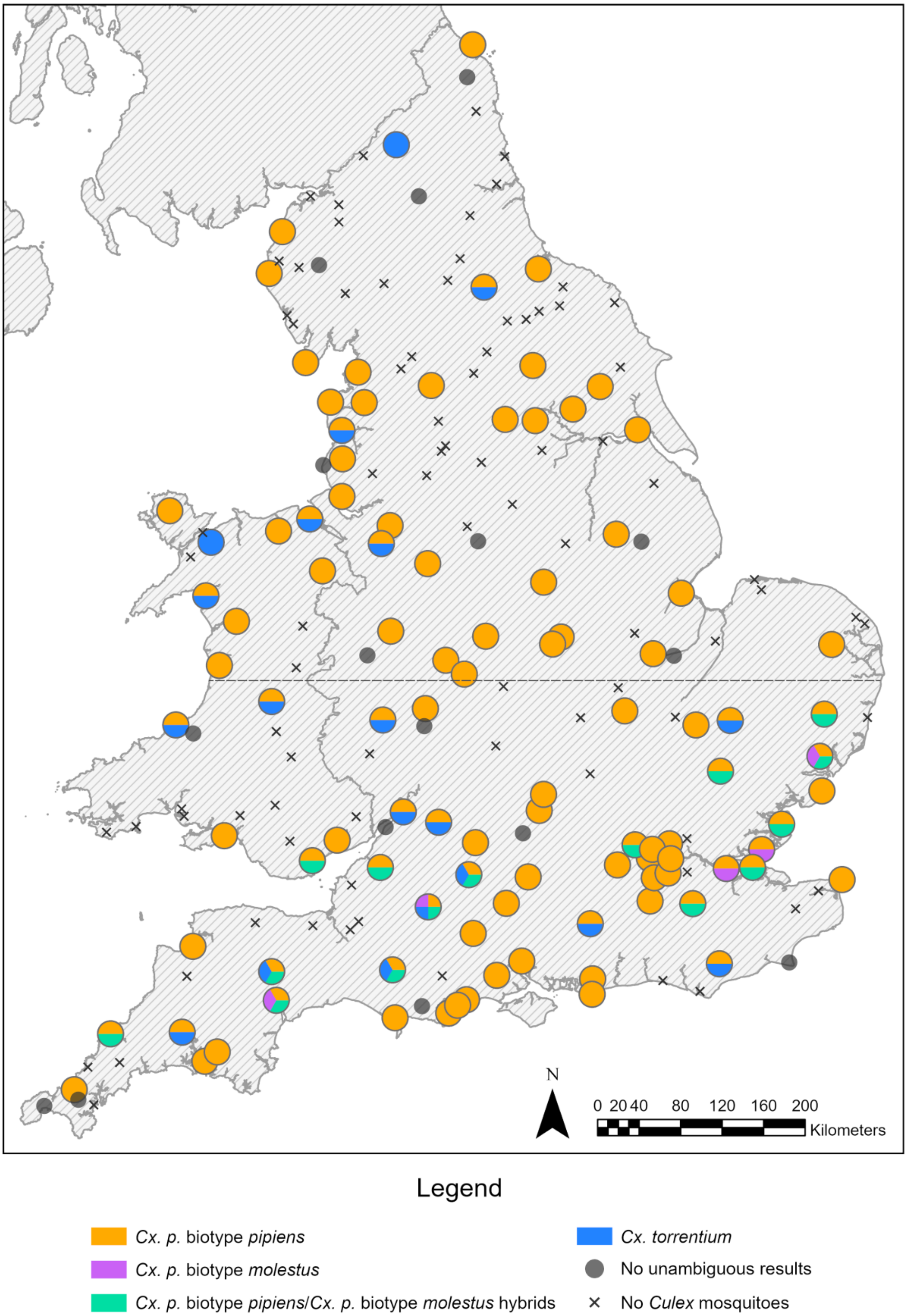

S2. A map showing an equal divide of each of the four *Culex* species found at each site, i.e. pure orange squares represent sites where only *Cx. pipiens* was found, whilst orange, pink, green and blue circles represent sites where all four of the *Culex* species were found. The dashed line represents the divide between trap sites visited by the northern and southern teams.

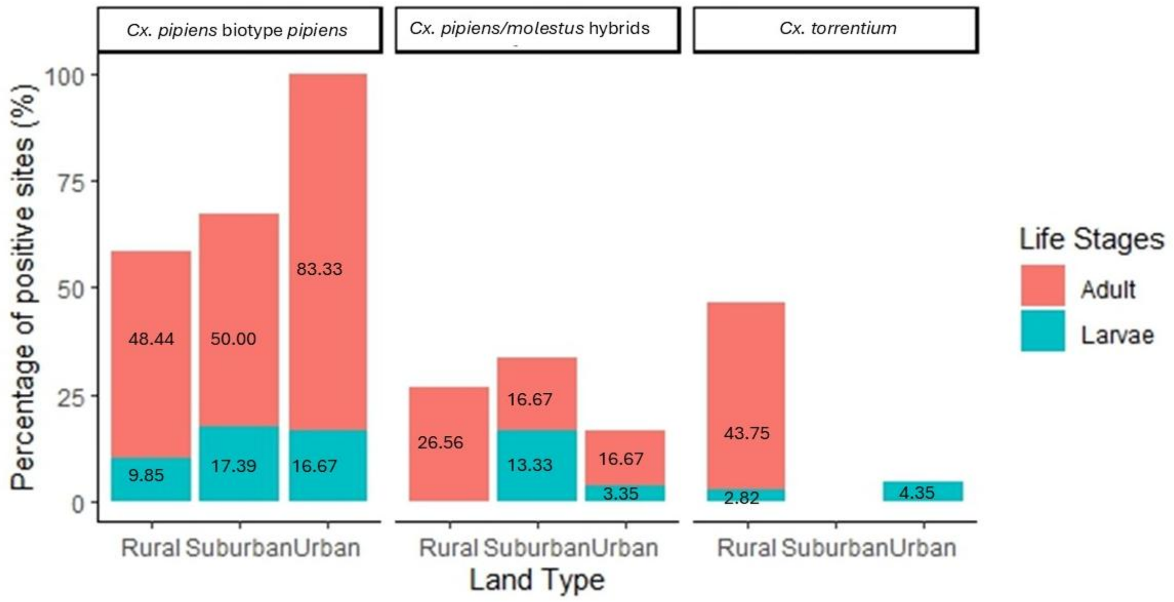

S3. A graph showing the percentage of each land type that was positive for adults or larvae of *Cx. pipiens* biotype *pipiens*, *Cx. pipiens/molestus* hybrids and *Cx. torrentium*. The data presented here is from the southern sampling.

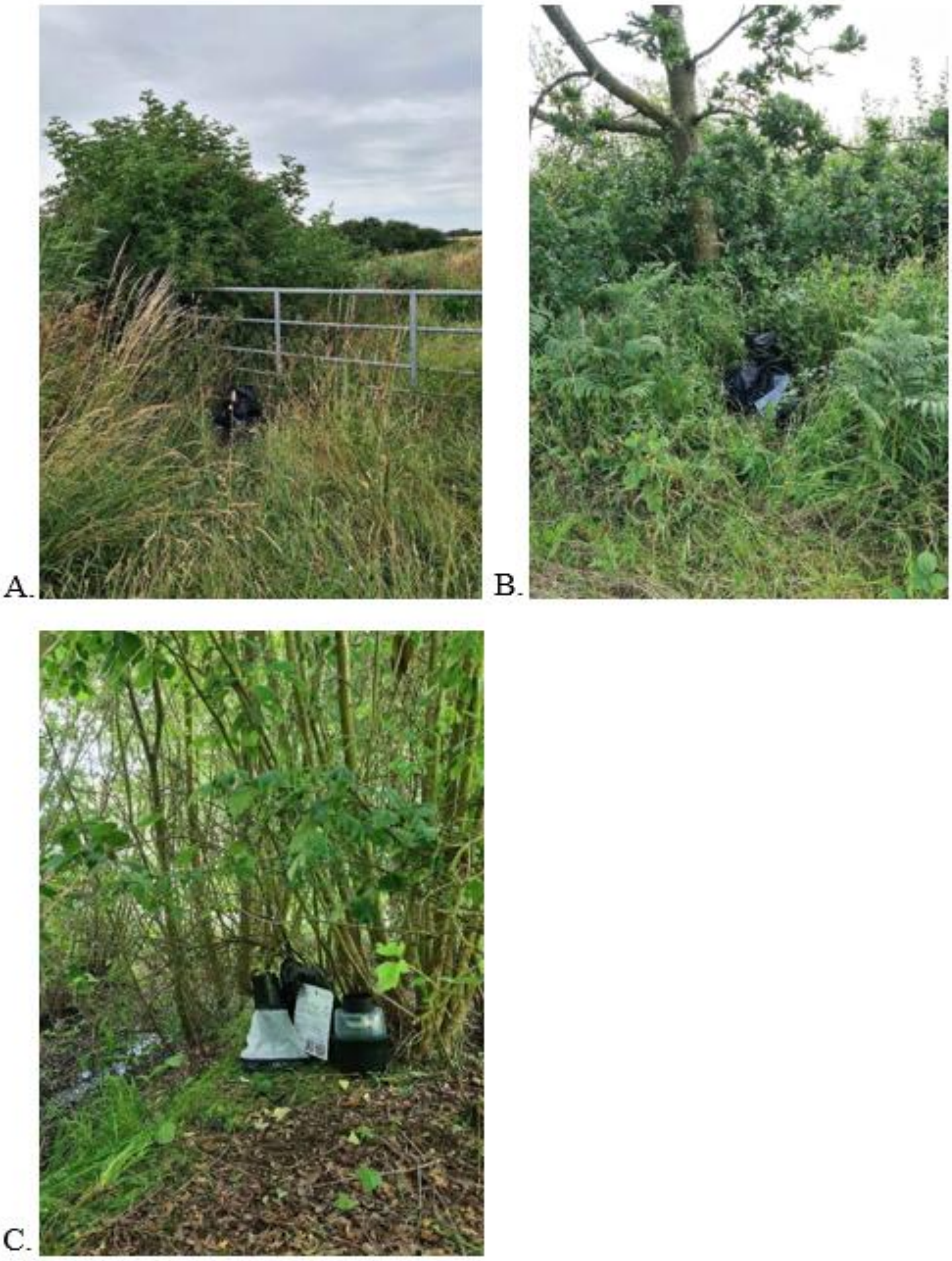

S4. Example photos of the BG-PRO and BG-GAT traps set up during the July 2023 *Culex* field collections. At all sites, both traps were placed within 1 m of each other. The traps were normally placed in sheltered areas. A. view of trap site from public footpath (the trap is visible just below centre), B and C. images of traps placed next to one another.
